# Minimising risk to thoracic surgical teams in an era of COVID-19: exploring possible preventative measures

**DOI:** 10.1007/s12055-020-01073-1

**Published:** 2020-11-18

**Authors:** Akshay Jatin Patel, Saifullah Mohamed, Edward Joseph Caruana, Babu Naidu

**Affiliations:** 1 Institute of Immunology & Immunotherapy, College of Medical and Dental Sciences, University of Birmingham, Vincent Drive, Edgbaston, Birmingham, UK; 2 Department of Thoracic Surgery, University Hospitals Birmingham NHS Trust, Birmingham, UK; 3 Department of Thoracic Surgery, University Hospital of Leicester, Leicester, UK; 4 NIHR Biomedical Research Centre, University of Nottingham, Nottingham, UK; 5 Institute of Inflammation & Ageing, College of Medical and Dental Sciences, University of Birmingham, Birmingham, UK

**Keywords:** COVID-19, Coronavirus, SARS-CoV-2, Thoracic surgery, Transmission

## Abstract

The demands of curtailing the impact of the coronavirus disease 2019 (COVID-19) global pandemic have disrupted the world’s ability to care for patients with thoracic pathologies. Those who undergo thoracic surgical therapeutic procedures are a high-risk category, likely to have impaired lung function but also high risk for exposing clinical teams to aerosolised viral loads. In light of this global pandemic, thorough pre-procedural planning, adequate personal protective equipment (PPE), experienced personnel and judicious anaesthetic and intra-operative measures will serve to be instrumental in ensuring positive patient outcomes whilst still protecting the safety of healthcare workers.

## Introduction

The novel coronavirus, now termed severe acute respiratory syndrome coronavirus 2 (SARS-CoV-2), has caused a significant global impact in the space of 4 months. This has had resounding effects on the ability to deliver adequate healthcare to all those who require it. The demands of curtailing the impact of the pandemic have disrupted the world’s ability to care for patients with thoracic pathologies. The ever increasing burden of the coronavirus disease 2019 (COVID-19) pandemic coupled with the finite amount of hospital resources has forced surgeons to prioritise cases and protect patients. At the same time, however, careful consideration must also be given to the hospital policies and procedures that need to be implemented in order to protect the healthcare teams from viral transmission. Numerous bodies from multiple cancer, surgical and research organisations have come together to form consensus statements on how we can mitigate the risk of transmission through optimisation of pre-operative, intra-operative and post-operative procedures [1]. This brief article seeks to provide an overview of the current guidance on how intra-operative precautions can be employed to make thoracic surgical procedures safer for all healthcare personnel involved.

## Comments

Therapeutic procedures on the lung during the era of COVID-19 automatically place clinical teams to a high risk of exposure from aerosolised viral loads. Procedures such as bronchoscopy, tracheostomy, double-lumen endotracheal tube placement, airway surgery, laparoscopy and lung surgery with post-operative parenchymal air leaks all pose a risk. Multi-disciplinary strategies should initially be put in place to triage patients to treatment pathways that are often alternative to surgery. These should be balanced against the COVID-19 status of the hospital, the level of hospital resources available and the COVID-19 trajectory of the hospital (i.e. not in rapid escalation phase) [1]. Those patients who do undergo surgery or allied thoracic surgical procedures must be carried out after judicious evaluation of the ‘patient benefit’ to ‘healthcare worker risk’ ratio.

Tracheostomy and bronchoscopy both have a continuing role in managing weaning from extended periods of mechanical ventilation during this pandemic. Working groups have determined that for tracheostomy, patient selection and timing of procedure are paramount [2]. The procedure be delayed until at least day 10 of mechanical ventilation and when there are signs of clinical improvement. Use of enhanced personal protective equipment (PPE) should be carried whenever possible in an appropriate location. Minimal use of diathermy is advocated with use of a smoke evacuator in the case of an open surgical technique. Pre-oxygenation, followed by a trial of apnoea in the intensive care unit (ICU), with an FiO_2_ of 1.0 and positive end-expiratory pressure of 5 cm H_2_O, in patients who are supine before tracheostomy to show physiological readiness to tolerate the procedure, with strategies to mitigate aerosolisation is also advised [2]. Little exists in the way of objective evidence to see if there is a tangible transmission impact from the implementation of these added precautions; however, Angel and colleagues [3] reported on the outcomes of 98 COVID-19-positive patients on mechanical ventilation undergoing a novel percutaneous dilational tracheostomy (PDT) procedure. The concurrent use of rigid bronchoscopy adjacent to the endotracheal tube provided good visualisation whilst reducing the risk of virus aerosolisation. The procedure was deemed safe and feasible with a low overall procedural complication rate (5.1%). At the time of reporting, 33% of patients had been weaned from mechanical support, 19% had their tracheostomy tube downsized and 8% were de-cannulated. Forty patients remained on full ventilator support. An 8-person healthcare team was involved in performing PDT on all the patients in this series; of these, 4 were formally tested for SARS-CoV-2, and none was positive. No person in the entire healthcare team developed any coronavirus-related symptoms. Furthermore, Mecham and colleagues [4] reviewed the literature for evidence on how to best perform tracheostomy in patients with potential COVID-19 infection. They concluded that adequate peri-procedural planning, enhanced provisions of PPE and careful anaesthesia are key to good patient outcome and ensuring the safety of all involved. Furthermore, timely execution of the procedure at the bedside would help to limit the number of personnel exposed. Wahidi and colleagues [5] provided six guiding statements on the role of bronchoscopy during the COVID-19 pandemic. Of these, one statement was evidence based (level 2C); the use of bronchoscopy to diagnose, stage or characterise known or suspected lung cancer in an area where COVID-19 transmission is present, should be carried out in a timely and safe manner. The remaining five consensus-based statements relate to pre-procedural COVID-19 patient testing and the provision and use of full PPE throughout.

During the conduct of thoracic surgery and elective lung resection, multiple guidance documents have been produced. Rakovich and colleagues [6] assimilated a multi-disciplinary working group in order to guide the conduct of elective lung cancer resections to reduce the risk of viral transmission through aerosolisation. Six time points were devised; strategies were devised to reduce the risk for aerosol at each time point (these are all detailed in Table 1). These strategies can help to revise and guide operating theatre protocols for the purposes of this pandemic and indeed similar situations in the future. Thornton and colleagues [7] developed a set of practice-based recommendations for airway management and lung isolation during thoracic surgery to mitigate against the risk of aerosolisation. Guidance is provided around tracheal intubation and extubation, conduct of lung isolation and single-lung ventilation as well as flexible bronchoscopy. When dealing with hypoxic episodes during single-lung ventilation, the authors recommend two-lung ventilation for critical hypoxia. However shunt-driven hypoxia can be overcome by administering oxygen to the dependent lung which is advised to be via a continuous positive pressure circuit with a built-in high-efficiency particulate air (HEPA) filter. Soma and colleagues [8] designed an 8-step operative checklist to reduce aerosolisation of secretions. The checklist was based on an example of paediatric laryngo-bronchoscopy for diagnostic and therapeutic purposes (foreign body removal). The steps start with notifying the surgical booking centre and on-call anaesthetist all the way through to surgical and anaesthetic completion and debrief. Measures are described at each step to reduce risk of aerosolisation. Intra-procedural measures include lowest acceptable gas flows for oxygenation and glass screens to protect surgical teams during visualisation through rigid bronchoscopy.

**Table 1:** Summary of working group guidelines

Author, date, journal and country, study type (level of evidence)	Patient group	Outcomes	Key results	Comments
Angel et al. (2020) [3] *Ann Thorac Surg*, USA Case series (level IV)	98 COVID-19+ve patients on mechanical ventilation ≥ 5 days undergoing a novel percutaneous dilational tracheostomy (PDT)	Safety and feasibility of PDT - Post-PDT bleeding	*N* = 5 (5.1%)	Modified PDT techniques involves placement of bronchoscope *alongside* endotracheal tube to mitigate risk of virus aerosolisation
		Early patient outcomes - Fully weaned post PDT	*N* = 32 (33%)	
		Early healthcare provider outcomes for COVID-19 symptoms and/or SARS-CoV-2 positive testing (*n* = 8, 4 of whom underwent formal testing with rtPCR assay testing)	*N* = 0	
Mecham et al. (2020) [4] *Laryngoscope*, USA Expert opinion (level V)	COVID-19-positive patients requiring tracheostomy	Tracheostomy	Bedside approach Bronchoscopy use with a percutaneous approach should be limited to decrease viral exposure Appropriate pre-procedural planning	Consensus on literature based on COVID-19 and 2003 SARS outbreak data
Porter et al. (2020) [9] *Br J Urol*, Global Panel Expert opinion (level V)	Minimally invasive surgical patients	Minimally invasive surgery (MIS) (laparoscopic, VATS, robotic)	Limit MIS to planned urgent or emergency procedures Reduce CO_2_ working pressure to the lowest acceptable level Suction residual CO_2_ from a patient into a closed filtration system using the smallest filter available	Appropriate Personal Protective Equipment (PPE) including N-95 masks should be made available to all healthcare workers involved. CO_2_ insufflation is optional in VATS thoracic surgery in view of the rigid chest wall.
Rakovich et al. (2020) [6] *Annals of Surgery*, Canada Expert opinion (level V)	Elective lung cancer resection patents	Intubation and Extubation	Seal off operating room until airborne contaminants have been removed Cough suppression strategies during extubation Double-lumen tube over bronchial blocker	Aerosol transmission risk divided into 6 time points with mitigating strategies for each time-point
		Lung isolation and patient positioning	Maintain closed circuit at all times Meticulous tube fixation and tight circuit connections	
		Access to chest	Interrupt ventilation when opening intercostal space	
		Conduct of surgery	Sparing use of energy devices Bipolar or ultrasonic devices preferred over standard cautery Dedicated suction devices for smoke Avoid CO_2_ insufflation Use of airtight thoracoscopic ports	
		Procedure termination and lung re-expansion	Use of tissue sealants for staple line integrity Avoid high ventilation pressures to prevent air leak Airtight closure of incisions and around chest tube Negative pressure lung re-expansion	
		Chest drainage	Electrostatic filter application to chest drainage systems	
Soma et al. (2020) [8] *Int J Pediatr Otorhinolaryngol*, Australia Expert opinion (level V)	Paediatric patients requiring laryngo-bronchoscopy for diagnostic purposes/foreign body retrieval	Intra-procedurally	Lowest gas flows to maintain oxygenation Use of a glass window plug through a rigid bronchoscope for visualisation	An 8-step operative team checklist was created to reduce aerosolisation of secretions during aerosol-generating procedures (AGP)
Thornton et al. (2020) [7] *Br J Anaesth*, UK Expert opinion (level V)	Thoracic surgery patients requiring lung isolation	Tracheal intubation	Pre-oxygenation to achieve an end-tidal F_E_O_2_ > 90% Ensure adequate neuromuscular blockade, as assessed with a peripheral nerve stimulator Release positive pressure within circuit with an adjustable pressure-limiting valve	
		Lung isolation	Place a high-efficiency particulate air (HEPA) viral filter prior to opening a double-lumen tube to the atmosphere Open to the atmosphere after allowing release of positive pressure within the lung through a HEPA filter	
		Flexible Bronchoscopy	Appropriate handling and designation of re-usable flexible bronchoscopes.	
Van den Eynde et al. (2020) [10] *J Robot Surg*, Belgium Expert opinion (level V)	Cardiothoracic robotic surgical patients	Robot-assisted cardiothoracic surgery	Minimise CO_2_ release Close port taps before insertion Attach a CO_2_ filter to one of the ports for smoke evacuation Deflate the thorax with a suction device prior to entering or removing material Avoid use of ultrasonic sealing and use lowest possible electrocautery energy One lung ventilation should be avoided if possible in COVID-19 diseased lungs particularly if this is associated with intolerable hypoxamia/hypercapnia in COVID-19 injured lungs	
Wahidi et al. (2020) [5] *Chest*, USA Expert opinion (level V)	Patients undergoing bronchoscopy	Bronchoscopy	Sparing use of procedure Defer all non-urgent cases in an area where COVID-19 transmission within the community is present PPE should be worn at all times	Systematic review and critical analysis of the literature.

In the world of Minimally Invasive and Robotic Surgery, similar working groups have been formed. Porter and colleagues [9] conducted a pan-specialty review in an effort to provide guidance on how to mitigate against the transmission of COVID-19 in minimally invasive surgery. The risk of COVID-19 transmission through CO_2_ insufflation during these procedures remains unclear; however, precautions should be undertaken to decrease exposure to surgical smoke, decrease production of surgical plume and filter any gaseous products (e.g. CO_2_) through pre-approved filters. Van den Eynde and colleagues [10] described guidance on the conduct of robot-assisted cardiothoracic surgical procedures in COVID-19 patients. Whilst the conduct of the surgery may be beneficial over traditional open methods in terms of length of post-operative stay and recovery, these must be balanced against the risk of viral aerosolisation which should be managed by judicious use of CO_2_ insufflation as well as high-energy electrocautery and ultrasonic devices.

## Conclusion

The COVID-19 pandemic has dramatically affected the conduct of healthcare practices globally. This has sparked the development of various guidelines to reduce the risk of viral transmission during thoracic surgery and allied specialties. In light of this global pandemic, there is a growing body of evidence, which is largely guideline, expert opinion and consensus driven, yet still represents the current best platform from which we can inform our precautions in the management of this uncertain threat. Thorough pre-procedural planning, adequate PPE, experienced personnel and judicious anaesthetic and intra-operative measures will serve to be instrumental in ensuring positive patient outcomes whilst still protecting the safety of healthcare workers.

## Data Availability

All data and material are publicly available.
